# Complex patterns of direct and indirect association between the transcription Factor-7 like 2 gene, body mass index and type 2 diabetes diagnosis in adulthood in the Hispanic Community Health Study/Study of Latinos

**DOI:** 10.1186/s40608-018-0200-x

**Published:** 2018-10-02

**Authors:** Lindsay Fernández-Rhodes, Annie Green Howard, Mariaelisa Graff, Carmen R. Isasi, Heather M. Highland, Kristin L. Young, Esteban Parra, Jennifer E. Below, Qibin Qi, Robert C. Kaplan, Anne E. Justice, George Papanicolaou, Cathy C. Laurie, Struan F. A. Grant, Christopher Haiman, Ruth J. F. Loos, Kari E. North

**Affiliations:** 1 Department of Epidemiology, UNC Gillings School of Global Public Health, University of North Carolina at Chapel Hill, 123 W Franklin St, Building C, Chapel Hill, NC USA; 2 Carolina Population Center, University of North Carolina at Chapel Hill, 123 W Franklin St, Building C, Chapel Hill, NC USA; 3 Department of Biostatistics, UNC Gillings School of Global Public Health, University of North Carolina at Chapel Hill, Chapel Hill, NC USA; 4 Department of Epidemiology and Population Health, Albert Einstein College of Medicine, Bronx, NY USA; 5 Department of Anthropology, University of Toronto at Mississauga, Mississauga, ON Canada; 6 Department of Medicine, Vanderbilt University Medical Center, Nashville, TN USA; 7 Biomedical and Translational Informatics Institute, Geisinger Health System, Danville, PA USA; 8 Epidemiology Branch, National Heart Lung and Blood Institute, Bethesda, MD USA; 9 Department of Biostatistics, School of Public Health, University of Washington, Seattle, WA USA; 10 Divisions of Human Genetics and Endocrinology, Children’s Hospital of Philadelphia Research Institute, Philadelphia, PA USA; 11 Department of Preventive Medicine, Norris Comprehensive Cancer Center, Keck School of Medicine, University of Southern California, Los Angeles, CA USA; 12 Charles R. Bronfman Instituted for Personalized Medicine, Icahn School of Medicine at Mount Sinai, New York, NY USA

**Keywords:** *TCF7L2*, Genetics, Obesity, Diabetes, Hispanic/Latinos

## Abstract

**Background:**

Genome-wide association studies have implicated the *transcription factor 7-like 2* (*TCF7L2*) gene in type 2 diabetes risk, and more recently, in decreased body mass index. Given the contrary direction of genetic effects on these two traits, it has been suggested that the observed association with body mass index may reflect either selection bias or a complex underlying biology at *TCF7L2*.

**Methods:**

Using 9031 Hispanic/Latino adults (21–76 years) with complete weight history and genetic data from the community-based Hispanic Community Health Study/Study of Latinos (HCHS/SOL, Baseline 2008–2011), we estimated the multivariable association between the additive number of type 2 diabetes increasing-alleles at *TCF7L2* (rs7903146-T) and body mass index. We then used structural equation models to simultaneously model the genetic association on changes in body mass index across the life course and estimate the odds of type 2 diabetes per *TCF7L2* risk allele.

**Results:**

We observed both significant increases in type 2 diabetes prevalence at examination (independent of body mass index) and decreases in mean body mass index and waist circumference across genotypes at rs7903146. We observed a significant multivariable association between the additive number of type 2 diabetes-risk alleles and lower body mass index at examination. In our structured modeling, we observed non-significant inverse direct associations between rs7903146-T and body mass index at ages 21 and 45 years, and a significant positive association between rs7903146-T and type 2 diabetes onset in both middle and late adulthood.

**Conclusions:**

Herein, we replicated the protective effect of rs7930146-T on body mass index at multiple time points in the life course, and observed that these effects were not explained by past type 2 diabetes status in our structured modeling. The robust replication of the negative effects of *TCF7L2* on body mass index in multiple samples, including in our diverse Hispanic/Latino community-based sample, supports a growing body of literature on the complex biologic mechanism underlying the functional consequences of *TCF7L2* on obesity and type 2 diabetes across the life course.

**Electronic supplementary material:**

The online version of this article (10.1186/s40608-018-0200-x) contains supplementary material, which is available to authorized users.

## Background

Hispanic/Latino adults in the United States (US) are disproportionally affected by obesity and it consequences such as type 2 diabetes (T2D) [1] and this disparity is widening as compared to non-Hispanic Whites [2]. The *transcription factor-7 like 2* gene (*TCF7L2*) was the first locus to be associated with T2D in genome-wide association studies (GWAS) and has been consistently associated with T2D [3, 4], *TCF7L2* (previously known as *TCF4*) encodes a transcription factor that is an effector of the Wnt signaling pathway [5]. Although the underlying biological mechanisms of *TCF7L2* remain unclear [6], the consistent association between the *TCF7L2* locus and T2D has been generalized to many diverse populations including Hispanic/Latinos [7, 8]. Indeed, the associated risk allele, rs7903146-T, harbored within the fourth intron of *TCF7L2* has the largest effect on T2D risk of all GWAS-identified T2D loci reported to date [8]. In Hispanic/Latinos each risk-allele has been associated with a 40% increased odds of T2D [7, 9].

The T2D-increasing allele at *TCF7L2* has also been associated with lower body mass index (BMI) [3, 10–12], resulting in a subsequent call for future research [13] given the strong epidemiologic correlation between increasing BMI and risk of T2D [14]. This association has been attributed to a T2D-related ascertainment bias, mainly due to the observation that the strongest and most significant *TCF7L2* associations with BMI are seen in T2D cases/controls, as compared to population-based studies [15–17].

There is mounting evidence of a complex biologic story for *TCF7L2*, explained in part by the bidirectional action of *TCF7L2* that may be cell, tissue or metabolically dependent [5]. Functional studies indicate that the rs7903146 variant may act in a cell or tissue-specific manner [18], by influencing alternative splicing of the *TCF7L2* [19–21], or by binding affinity of complex transcriptional machinery at an open chromatin region specific to human pancreatic islets [22–25] to modulate pancreatic islet cell insulin production and secretion [17], action in adipose tissue [26], hepatic glucose output [27] or intestinal tissue differentiation [28]. Observational studies indicate that the T2D risk allele at *TCF7L2* associates with decreases insulin secretion [29–31]. Thus, we may expect individuals with the T allele have lower BMI values on average, and perhaps a differential pattern of insulin resistance.

Due to the mounting evidence on potential selection bias and the multi-faceted action of *TCF7L2* variation on insulin and glucose biology [5, 6, 18], we aimed 1) to replicate the multivariable association between *TCF7L2* T2D risk alleles and lower BMI in a population-based study of US Hispanic/Latinos accounting for key covariates, and 2) to model the structured pathways between rs7903146, at *TCF7L2*, BMI over time, and age of diabetes diagnosis. We performed these analyses in 9031 self-identified Hispanic/Latino adults (21–76 years of age at examination) residing in four US urban centers, who consented to genotyping and provided weight history and T2D diagnosis information the Hispanic Community Health Study/Study of Latinos (HCHS/SOL) baseline examination (2008–2011).

## Methods

### Study participants

We used data from the HCHS/SOL study, a multi-center, longitudinal, household-based cohort study of 16,415 Hispanic/Latino adults, aged 18–76 years in 2008–2011, who were sampled using a two-stage probability design from four US urban communities (The Bronx, NY; Chicago, IL; Miami, FL; San Diego, CA), as described previously in detail [32, 33]. Briefly, the complex sampling design allowed researchers to 1) over-sample individuals ≥45 years of age who were most likely to experience cardiometabolic disease outcomes either by the baseline examination or during follow up, while 2) capturing the varied socioeconomic and demographic composition of Hispanic/Latino households (as per the 2000 Census block group proportion of residents ≥25 years old with at least a high school education and the proportion Hispanic/Latino residents) and efficiently estimating cardiometabolic disease across the four Hispanic/Latino communities under study. Centrally-trained study personnel conducted the screening and baseline examinations in either English or Spanish based on participant preference.

### Body mass index

As part of the HCHS/SOL baseline examination [32], current body weight was self-reported (in whole lb. or kg) and measured (to a tenth of a kg) and height was measured (to whole cm) on participants who were able to stand on both feet. As described previously [34], the accuracy and reliability of the self-reported weights were good (mean difference_self-report–measured_ = 0.23 kg, r^2^ = 0.97; inter-rater reliability coefficients, 0.93 and 0.97). Waist circumference was measured in cm at the umbilicus using a tape measure, and body fat percentage estimated by a Tanita Body Composition Analyzer.

Additionally, a weight history questionnaire was used to collect self-reported body weights (in whole lb. or kg, while not pregnant) at 21, 45, and 65 years of age, for individuals 21 years or older at baseline. If participants indicated that they could not remember their exact weight, personnel were instructed to inquire about their best guess. The quality control procedures and data cleaning are described in the Appendix. We converted each weight from the weight histories to kg and rounded each weight to the whole unit, to eliminate measurement error by unit of report (e.g. lb. or kg).

We excluded all weights from women who reported currently being pregnant at baseline or individuals with limb amputations that otherwise did not limit their ability to stand (Additional file 1: Figure S2 and Table S1). Using measured height at baseline, we calculated two baseline BMI measures (kg/m^2^) and up to three BMIs from the weight histories of individuals at least 21 years of age (corresponding to 21, 45, and 65 years). We further excluded any BMI that was less < 16 or > 70 kg/m^2^. As measured height is an imperfect proxy of an individual’s height at various times in the past, all models of BMI from the weight histories (at 21, 45, and 65 years) also accounted for the age at baseline as a measure of age at time of recall.

### Type 2 diabetes assessment

HCHS/SOL participants were asked to bring in the medications they were currently taking, during the baseline examination. Individuals were also asked to report if a “doctor ever said that you have diabetes (high sugar in blood or urine)” and the age when this diagnosis was received. Participants were asked to fast overnight (> 8 h) and their glucose was measured in the entire sample, and 2-h post-oral glucose tolerance tests was measured among those who reported never having received a diabetes diagnosis. Impaired fasting glucose among non-diabetics was defined as a fasting glucose 100-125 mg/dL or 140-199 mg/dL after oral glucose challenge. We used the American Diabetes Association definition to identify T2D cases at examination based on fasting glucose (≥126 mg/dL), an oral glucose tolerance test (OGTT, ≥200 mg/dL), percent Glycated Hemoglobin (HbA1C ≥6.5%), or diabetes medications [35]. Controlled diabetes was further defined as % HbA1C < 7%.

Type of diabetes was not reported in HCHS/SOL. Therefore, we used information on age at diabetes diagnosis to create age period-specific T2D diagnosis indicators. If an individual was younger than 45 or 65 years at the baseline examination, then the classification of T2D diagnosis of the incomplete age period was set to missing (e.g. for a 50-year-old, T2D diagnosis for the period of 22–45 years could be yes/no, but would be set to missing for 46–65 years).

### Genetic information

Venous blood samples were collected and for all fully consenting participants (i.e. those agreeing to genotyping and sharing of information with HCHS/SOL investigators, those not affiliated with HCHS/SOL, and specialized laboratories) and were analyzed using the MetaboChip (Illumina, Inc., San Diego, CA) (*N* = 12,209 or 74% of the cohort). The MetaboChip array contains approximately 200,000 single nucleotide polymorphisms (SNPs) at 257 genomic regions previously associated with cardiometabolic traits, including the *TCF7L2* region that includes 258 SNPs across over 76,159 bp [36]. HCHS/SOL participants used in this study were genotyped at the Human Genetics Center of the University of Texas-Houston (Houston, TX) and passed person-level quality control filters (< 95% call rate, sex discordance or duplicate).

Based on previous trans-ethnic fine-mapping studies with T2D [37] and BMI [15], we selected rs7903146 as our presumed functional variant of interest at *TCF7L2* as it was in strong linkage disequilibrium with several other variants in the area (Additional file 1: Figure S1). In HCHS/SOL, this SNP also had satisfactory quality control measures [38], was in Hardy-Weinberg-Equilibrium (*P* value = 0.10), and available in the entire sample that passed genetic quality control procedures (*n* = 12,117). We created an additive score of the number of T2D risk alleles [7, 8] per individual at rs7903146 (e.g. *CC* = 0, *CT* = 1, *TT* = 2). To aid in the interpretability of adjustments for population stratification, we adjusted for continental ancestry proportions, which as reported on previously [39] were designed to represent four a priori-selected ancestral populations using a supervised analysis (*K* = 4; unrelated 1000 Genomes references representing European: CEU; African: YRI; Northern: MXL; Caribbean/Southern Native American Ancestry: PUR, CLM) in the program ADMIXTURE [40] on a pruned set of more than 45,000 MetaboChip SNPs in low linkage disequilibrium in our sample (r^2^ < 0.5). Lastly, we also adjusted for the ‘genetic analysis group’ variable from the multidimensional clustering of self-reported Hispanic/Latino background and principal components from genome-wide data on a majority-overlapping sample of 12,803 HCHS/SOL participants (> 99% call rate), as described previously [39].

### Statistical analyses

**Fig. 1:**
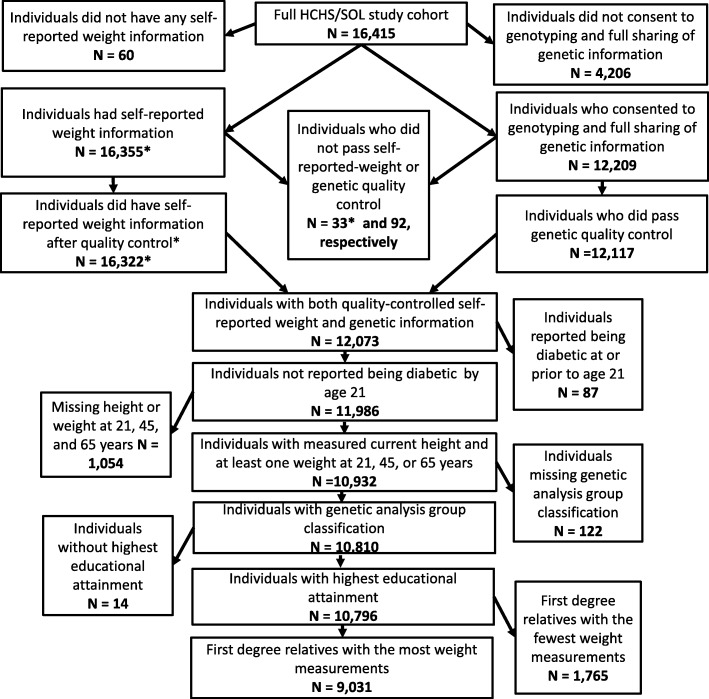
Flowchart of Quality Control and Exclusions Applied to the Hispanic Community Health Study/Study of Latinos (HCHS/SOL) Cohort of 16,415 Individuals (18–76 years), which Resulted in a Final Analytic Sample of 9031 Hispanic/Latino Adults (21–76 years), Which Are Detailed Further in the Additional file 1: Table S1 and Figure S2*

As shown in Fig. 1, of the entire HCHS/SOL baseline cohort of 16,415 participants, 16,322 individuals had self-reported and measured weight values that passed quality control (additional information provided as part of Additional file 1: Figure S2 and Table S1). Of the 12,209 individuals providing their full informed consent for genotyping and data sharing, 12,117 passed genetic quality control, as described above. The union of these two quality controlled data sets included 12,073 individuals (Fig. 1), from which we excluded 87 individuals who reported diabetes diagnosis prior to 22 years of age, to restrict our analysis to those for which a diabetes diagnosis was more likely to be T2D, and 1054 individuals that did not have both a measured current height or at least one self-reported weight at 21, 45 or 65 years and who were therefore unable to contribute to our structural equation modeling. Individuals with missing covariate information, such as missing genetic analysis group (*N* = 122) or information on their highest education level achieved (categorized as less than or at least a high school diploma or equivalency) (*N* = 14), were excluded. Lastly, as described previously in HCHS/SOL we used an identity-by-descent analysis in PLINK [41] to identify close relatives (e.g. 0.35 < π < 0.98) [42], and exclude the individual in each pair with the least weight measurements (*N* = 1765). A total of 9031 individuals remained in the final analytic dataset used for all analyses, and we described their characteristics using descriptive statistics such as means, 95% confidence intervals (CIs), and frequencies.

Next, we modeled the association between the additive number of rs7903146-T alleles with multiple BMI measures using multivariable models (e.g. measured and self-reported BMI at baseline, as well as BMI for ages 21, 45 and 65 years), controlling for age at examination, sex, educational attainment, admixture proportions, and genetic analysis group. As an exploratory analysis, we also examined the multivariable associations with the measured BMI stratified by previous diabetes diagnosis, as well as glucose tolerance and diabetic medication at examination.

Using structural equation models*,* we then examined an a priori-specified set of pathways (Additional file 1: Figure S3) between the additive number of rs7903146-T alleles, BMI (at age 21, 45 and 65), and self-reported T2D status (between ages 22–45 and between ages 46–65). BMI was assumed to be directly associated with T2D status in the period immediately after the BMI measurement (between ages 22–45 or 46–65). Similarly, T2D status during the period of time immediately preceding a given BMI (e.g. T2D between 22 and 45 years and 45-year-old weight) was assumed to be directly associated with the BMI at that time. Direct pathways between rs7903146 to BMIs and T2D measurements were also included. BMI at the previous age was assumed to be directly related to BMI at the following age. Age at baseline examination (age at time of recall), sex, education level, admixture proportions and genetic analysis group, and were included in all pathways to BMI and T2D.

All analyses accounted for the HCHS/SOL complex sampling design, including primary sampling unit, strata and sampling weights, yielding valid estimates of the disease distribution in the source population. Descriptive statistics were estimated using SAS 9.3 (Research Triangle Park, NC). All multivariable and structural equation models were estimated using Mplus 7.11 software [43], using full-information maximum likelihood methods to account for missing outcome data. Additionally, we identified our analysis subpopulation (*N* = 9031), or the subpopulation of interest in any stratified models, and used the complex sampling information on the entire cohort in the variance calculations to ensure valid estimates for the source population of HCHS/SOL.

## Results

Our weighted sample included women (50%) and men, an average age of 44 years at baseline examination (Table 1). Five percent of those who were at least 45 years old (unweighted *n* = 5605) received a T2D diagnosis by age 45 (Table 1). In the subsample of participants who were at least 65 years of age (*n* = 729), 23% reported received a diabetes diagnosis by age 65, with 3% being diagnosed by age 45 and 20% diagnosed between ages 46–65 years. Average BMIs increased across age of recalled weight (24 kg/m^2^ at 21 years to 29 kg/m^2^ at 65 years).

**Table 1:** Weighted Descriptive Statistics of Hispanic Community Health Study/Study of Latinos Baseline (2008–2011) Analytic Sample of 9031 Adults (21–76 years) from Four Urban United States Centers: The Bronx, NY; Chicago, IL; Miami, FL; San Diego, CA

**Unweighted Analytic Sample Size and Weighted Frequency or Means (95% Confidence Interval)**		
Female (%)	*n* = 5187	50%
Mean (95% CI) Age at Baseline Examination	*n* = 9031	43.67 (43.12, 44.21)
Has High School Diploma or Equivalency (%)	*n* = 5768	69%
Genetic Analysis Group (%)	South American (*n* = 676)	6%
	Central American (*n* = 1069)	8%
	Cuban (*n* = 1764)	27%
	Dominican (*n* = 797)	9%
	Mexican (*n* = 3169)	33%
	Puerto Rican (*n* = 1556)	17%
rs7903146 Genotype Frequency (%)	*CC* (*n* = 5040)	55%
	*CT* (*n* = 3373)	38%
	*TT* (*n* = 618)	7%
Mean (95% CI) BMI (kg/m^2^)	At 21 Years (*n* = 8759)	23.77 (23.61, 23.93)
	At 45 Years (*n* = 5605)	27.48 (27.30, 27.66)
	At 65 Years (*n* = 729)	28.99 (28.46, 29.53)
% with Self-Reported Diabetes Diagnosis	Between the Ages of 22 and 45 Years	5%
	Between the Ages of 46 and 65 Years	23%

Abbreviations: *BMI* body mass index, *95% CI* 95% confidence interval

The number of T2D-risk alleles at rs7903146 associated with an increase in T2D prevalence by 7% (*P* value = 0.0002) and decreased obesity prevalence by 3–5%, based on either the use of measured or self-reported weights (*P* value < 0.04, Table 2). Mean BMI and waist circumference at examination showed similar quantitative decreases by 0.5–0.6 kg/m^2^ and 1.1 cm as the number of T2D-risk allele increased (*P* values< 0.1). Additionally, among the subsample without a past diagnosis of T2D, at examination mean OGTT glucose levels increased by (4 mg/dL difference; *P* value = 0.06) and HOMA Index of Beta Cell function decreased (12 point difference; *P* value = 0.07). Other T2D-related measures, such as fasting glucose, insulin, and HbA1C exhibited similar trends across genotypes, but these trends were not statistically significant (*P* values ≥ 0.1). Further stratification of BMIs by T2D status/age at examination suggested that both increased age and T2D status corresponded to higher average BMIs, regardless of the timing of T2D diagnosis (Table 3). The subset of participants > 65 years at examination self-reported weights corresponding to a mean BMI increase of 2.8 kg/m^2^ between 45 and 65 years of age among those without T2D at baseline, and of up to 3.4 kg/m^2^ among those that were diagnosed with T2D after age 65.

**Table 2:** Anthropometric Measures (Body Mass Index; Weight; Height; Waist Circumference; Overall and Abdominal Obesity; Percentage Body Fat), Fasting Insulin and Glucose, Post-Oral Glucose Tolerance Test Response, and Diabetic Control Characteristics Weighted Means (Standard Deviations) or Frequencies across rs7903146 Genotypes (*n* = 9031) at the Hispanic Community Health Study/Study of Latinos Baseline Examination

	*CC*	*CT*	*TT*	*P*-value
**Unweighted total analytic sample size**	*n* = 5040	*n* = 3373	*n* = 618	
Measured BMI^a^ (kg/m^2^)	29.74 (0.17)	29.26 (0.13)	29.21 (0.28)	0.06
Self-Reported BMI^a^ (kg/m^2^)	29.81 (0.17)	29.34 (0.13)	29.23 (0.27)	0.05
Measured Weight (kg)	80.09 (0.52)	78.71 (0.39)	78.58 (0.79)	0.1
Self-Reported Weight (kg)	80.29 (0.53)	78.94 (0.39)	78.69 (0.76)	0.1
Measured Height (cm)	163.93 (0.18)	163.83 (0.19)	163.94 (0.32)	0.9
% Overall Measured Obesity (≥30 kg/m^2^ Measured BMI)	42.43%	37.89%	38.55%	0.03
% Overall Self-Reported Obesity (≥30 kg/m^2^ Self-Reported BMI)	43.29%	38.85%	37.66%	0.03
Waist Circumference (cm)	98.41 (0.40)	97.37 (0.31)	97.34 (0.65)	0.09
% Abdominal Measured Obesity (≥120 cm for men; ≥88 cm for women)	56.1%	55.2%	57.1%	0.7
% Body Fat	33.58 (0.21)	32.96 (0.19)	33.18 (0.4)	0.08
% Identified as Diabetic Before or at Baseline Examination^b^	14.77%	18.24%	21.01%	0.0002
**Unweighted non-diabetic subsample**	*n* = 4107	*n* = 2586	*n* = 454	
Fasting Insulin (mU/L)	11.98 (0.2)	11.96 (0.19)	11.53 (0.47)	0.7
Fasting Glucose (mg/dL)	93.78 (0.18)	94.12 (0.23)	94.67 (0.51)	0.2
HOMA Index of Beta Cell Function	145.17 (2.59)	140.81 (2.33)	133.28 (4.81)	0.07
HOMA Index of Insulin Resistance	2.82 (0.05)	2.84 (0.05)	2.75 (0.12)	0.8
post OGTT Insulin (mU/L)^c^	78.06 (1.81)	81.09 (1.84)	75.39 (3.2)	0.2
post OGTT, Glucose (mg/dL)^c^	112.25 (0.78)	114.23 (0.85)	115.85 (1.6)	0.06
Glycated Hemoglobin (mmol/mol)	35.77 (0.1)	35.99 (0.1)	35.72 (0.22)	0.2
% Glycated Hemoglobin	5.42 (0.01)	5.44 (0.01)	5.41 (0.02)	0.2
**Unweighted diabetic** ^**b**^ **subsample**	*n* = 933	*n* = 787	*n* = 164	
Glycated Hemoglobin (mmol/mol)	57.55 (1.25)	57.79 (0.94)	60.06 (2.06)	0.6
% Glycated Hemoglobin	7.42 (0.11)	7.44 (0.09)	7.64 (0.19)	0.6
% Controlled Diabetes (< 7% Glycated Hemoglobin)	26.1%	31.2%	35.3%	0.1

All weighted means and standard deviations (or percentages) for anthropometric measures (weight, height, body mass index, waist circumference, fat percentage, overall and abdominal obesity) were estimated from regression models, which accounted for the complex sampling design and age, sex, and ancestry proportions. Additionally, all other weighted means and standard deviations (or percentages) were adjusted for body mass index (BMI) at examination. rs7903146 genotypes were modeled dis-jointly (i.e. no additive model was assumed)

^a^Measured and self-reported BMI values at baseline were based off of measured weight and height, and self-reported weight and measured height, respectively

^b^The diabetes subsample included individuals reporting having received a previous diabetes diagnosis at baseline examination, or being identified as diabetic at the baseline examination

^c^2-h Oral Glucose Tolerance Test (OGTT) was conducted in only individuals who did not report having had a previous diabetes diagnosis

**Table 3:** Weighted Mean Body Mass Indices at 45 and 65 years by Categories of Baseline Examination and Diabetes Diagnosis Ages in the Hispanic Community Health Study/Study of Latinos among Participants > 45 Years of Age (*n* = 5643)

	Unweighted Sample Size	BMI (95% CI) at Age 45 (kg/m^2^)	BMI (95% CI) at Age 65 (kg/m^2^)
**Between 46 and 65 Years of Age (** ***n*** ** = 4,914)**			
No Diabetes Diagnosis Reporting at Baseline Examination^a^	*n* = 4094	27.49 (27.29, 27.69)	–
Diagnosed Between 22 and 45	*n* = 318	30.36 (29.53, 31.19)	–
Diagnosed Between 46 and Age at Examination	*n* = 502	29.30 (28.68, 29.93)	–
**Between 66 and 76 Years of Age (** ***n*** ** = 729)**			
No Diabetes Diagnosis Reported at Baseline Examination^a^	*n* = 502	25.67 (25.16, 26.18)	28.43 (27.72, 29.14)
Diagnosed Between 22 and 45	*n* = 26	26.51 (25.03, 27.99)	29.71 (27.56, 31.86)
Diagnosed Between 46 and 65	*n* = 153	27.75 (27.06, 28.45)	30.65 (29.53, 31.77)
Diagnosed Between 66 and Age at Examination	*n* = 48	25.89 (24.71, 27.07)	29.32 (27.58, 31.07)

Abbreviations: *BMI* body mass index, *95% CI* 95% confidence interval

^a^This categorization includes individuals who were first diagnosed at the baseline clinic visit

### Multivariable association analyses

We observed an association between the rs7903146 T2D-increasing allele and lower BMI, after adjustment for age, sex, education level, admixture proportions, and genetic analysis group. Specifically, we found that each T allele associated with lower BMI at examination (21–76 years), based on either measured or self-reported weight (Table 4). As described previously [15], we also observed significant inverse associations between each rs7903146-T allele and BMI (− 0.37 kg/m^2^, 95% CI: -0.69, − 0.06). Additionally, we also observed non-significant multivariable associations between rs7903146-T and lower BMIs at 21, and 45 years of age, and non-significant increases in BMI at 65 years of age.

**Table 4:** Adjusted Parameter Estimates^a^ between rs7903146-T and Body Mass Indices^b^ at the Hispanic Community Health Study/Study of Latinos Baseline Examination Representing 9031 Individuals (21–76 Years of Age), and at Several Ages Across the Lifecourse

	Sample Size^c^	Estimated Change in kg/m^2^ (95% CI) per T2D risk allele	*P*-value
Measured BMI at Examination	*n* = 9012	−0.37 (− 0.69, − 0.06)	0.019
Self-reported BMI at Examination	*n* = 8921	−0.38 (− 0.69, − 0.08)	0.015
Self-reported BMI at 21 Years	*n* = 8759	−0.20 (− 0.45, 0.04)	0.109
Self-reported BMI at 45 Years	*n* = 5605	− 0.18 (− 0.43, 0.06)	0.134
Self-reported BMI at 65 Years	*n* = 729	0.01 (− 0.82, 0.84)	0.980

Abbreviations: *BMI* Body mass index, *T2D* Type 2 diabetes, *95% CI* 95% confidence interval

^a^Corresponding to the additive number of T2D risk alleles at *TCF7L2* (rs7903146-T) and then adjusted for age, sex, education level, genetic ancestry group and ancestry proportions

^b^Body mass index (BMI) was based on self-reported or measured weight in kg, and divided by squared measured height at examination in meters (kg/m^2^)

^c^Unweighted sample size differences were a result of individuals missing certain weight measurements. For example, only individuals that were had reached 21, 45 or 65 years of age were asked to provide their weight for that particular age

Using data from the baseline examination, we also ran these BMI models stratified by previous diabetes diagnosis, glucose tolerance and medication status at the baseline examination (Additional file 1: Table S2). Weaker effects per allele on BMI were estimated among participants who reported having diabetes at examination as compared to those without diabetes, regardless of the use of measured or self-reported BMI at examination (− 0.30 to − 0.17 versus − 0.45 to − 0.48 kg/m^2^ per allele). Compared to our significant protective effect on BMI among all individuals without a prior diabetes diagnosis (− 0.45 kg/m^2^ per allele), the subset of individuals who had impaired fasting glucose or undiagnosed T2D at the examination (*N* = 4284) appeared to have an even stronger protective estimated effect on BMI (− 0.66 and − 0.85 kg/m^2^, respectively). No significant effects were seen for individuals taking diabetes medication at examination.

### Structured association analyses

**Fig. 2:**
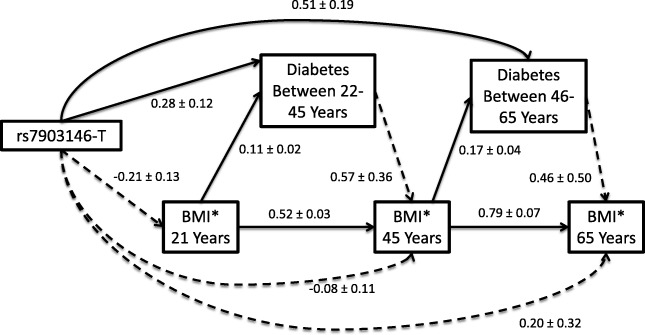
Illustration of structured pathways (effect estimates and standard errors) between the additive number of rs7903146-T alleles, diabetes, and Body Mass Index (BMI), showing paths with *P* values < 0.05 in solid lines

In a structural equation model, we noted that each T allele at rs7903146 was directly associated with a 1.32 (95% CI: 1.05, 1.67) higher odds of T2D diagnosis between the ages of 22 and 45 years, and a 1.67 (95% CI: 1.15, 2.42) higher odds of T2D diagnosis between 22 and 65 years of age. We did not find any significant direct associations between the rs7903146-T and BMI at any age; however, the direction of estimated effect was inverse on BMI at 21 and 45 years (Fig. 2). Furthermore, we found no evidence of indirect associations between rs7903146 and either BMI or T2D at any time point (Additional file 1: Table S3). Similarly, the indirect association between rs7903146 and BMI at 45 and 65 years, as mediated through a previous T2D diagnosis, was negative but non-significant (Additional file 1: Table S4).

## Discussion

In this study we successfully replicated the previously-reported association between T2D risk alleles at *TCF7L2* (rs7903146-T) and decreased BMI [3, 10–12], within a population-based cohort of US Hispanic/Latino adults of multiple background groups living in four urban communities (21–76 years of age at examination). We also observed consistently protective, albeit non-significant, associations on BMI at 21 and 45 years. In contrast, among the subset of individuals 65 years or older, the non-significant association between T2D-risk variants and BMI at 65 years of age was positive.

Next, we employed a structural equation model to examine the direct and indirect pathways between rs7903146, T2D and BMI, which revealed that this suggestive protective effect between T2D-risk variants and BMI at 21 and 45 years of age remained even after controlling for earlier BMI. These results collectively suggest that there may be a persistent independent protective effect of *TCF7L2* T2D risk alleles on BMI across most of adulthood. In contrast to a previous cross-sectional study of 1235 Hispanic/Latinos, which estimated a larger effects of T2D-risk alleles at *TCF7L2* on BMI by adjusting for concurrent T2D status (− 0.3 to − 1.1 kg/m^2^ for rs12255372-T) [12], our large and diverse study of US Hispanic/Latinos estimated more modest effects of T2D-risk alleles on BMI (− 0.4 kg/m^2^ for rs7903146-T; unadjusted for T2D status) and leveraged information on weight and T2D histories collected during the HCHS/SOL baseline examination to further decompose the complex relationships between prior BMI and T2D (Effect of each T2D-risk allele on BMI ranged from − 0.2 to 0.2 kg/m^2^ at 21 and 65 years of age, respectively).

Our findings shed light on the two predominant hypotheses put forth to explain the inverse direction of association between T2D and BMI at *TCF7L2,* as captured by variation in rs7903146. First, it has been suggested that case ascertainment bias [17] may drive the association of *TCF7L2* T2D risk alleles and lower BMI, as previous GWAS have shown attenuated effects of *TCF7L2* on BMI among population-based samples as compared to the effect sizes in samples of T2D cases [15]. Specifically, collider stratification may bias the *TCF7L2-*BMI association downwards when the ratio T2D cases to controls has been distorted to over-represent cases, or cases with more favorable insulin resistance profiles [44]. The active HCHS/SOL community engagement, household sampling, and location of clinic sites in the local community all served to minimize selection bias.

The consistent negative association between T2D risk alleles and BMI in early and mid-adulthood seen in this and previous work [3, 10, 11] may point to another explanation. A growing body of literature implicates pleiotropy at *TCF7L2* in both T2D and BMI [5]. We observed protective associations on BMI at 21 and 45 years of age, which were not explained by accounting for indirect pathways through T2D or earlier BMI in our structured modeling. This work leverages detailed weight history data to provide further evidence for a complex mechanism underlying *TCF7L2* action across the life course that may explain its associations with both T2D and BMI [3, 10–12], or the apparent statistical interaction between *TCF7L2* genotype and adiposity on T2D related traits seen in previous cross-sectional studies of US Hispanic/Latinos [31]. Yet, clearly future functional or longitudinal analyses in population-based samples are required to substantiate our study’s findings.

Herein, we were also able to explore for the first time to our knowledge, what might be the direct effect of T2D diagnosis on subsequent BMI in the context of *TCF7L2* genetic effects. The receipt of a T2D diagnosis between 22 and 45 years of age was significantly associated with an average increase in BMI at 45 years, as compared to those that never received a diagnosis during this time (Fig. 2). We did observe a similar, but non-significant association of T2D diagnosis between 46 and 65 years on BMI at 65 years. This indicates that the possible impact of pre-diagnosis metabolic dysfunction, T2D-related lifestyle counseling, or medical intervention also does not fully explain the apparent negative association between the *TCF7L2* T2D risk allele and BMI [15]. This was further supported by our non-significant *TCF7L2* associations on BMI at the examination among T2D individuals concurrently taking medications (Additional file 1: Table S2).

This current analysis is additionally strengthened by its focus on adults of varied Hispanic/Latino backgrounds [45]. Our sampling weights accounted for non-response and our statistical modeling approach also allowed us to account for missing data under the assumption of non-informative missingness and to base our variance calculations on information on the full population-based sample. In our dataset, missingness for age-specific BMIs was primarily determined by one’s age (BMI at 45 and 65 years of age would be missing for a 35-year-old participant).

Even though a previous study, which did not genotype rs7903146 directly, has posited that their best marker SNP at *TCF7L2* (rs12255372, r^2^ = 0.7 in AMR with rs7903146) may capture a secondary BMI signal in Hispanic/Latinos [12], subsequent trans-ethnic fine-mapping studies of BMI and T2D including diverse Hispanic/Latino samples [8, 46] and large Hispanic/Latino studies have not supported the presence of multiple signals [47]. This gives us confidence that rs7903146, the lead variant for the single T2D signal observed in HCHS/SOL [48], is the best available SNP marker to simultaneously investigate allelic effects on BMI and T2D diagnosis within a structural equation modeling framework. Nonetheless, we do acknowledge that our current results do not capture all possible sources of pleiotropy at the* TCF7L2* locus, which warrants further study.

Our structural equation results are also limited by our reliance on self-reported age of diabetes diagnosis, instead of repeated quantitative measures of T2D or its successful control. Among Hispanic/Latinos 15–19 year old, less than two thirds of diabetes cases may be Type 1, but the type distribution of cases steadily trends towards more T2D cases into early adulthood [49]—a period captured in HCHS/SOL. For this reason, we excluded a small number of individuals reporting early onset (< 22 years, *N* = 87) of diabetes. In HCHS/SOL, an additional *N* = 344 individuals reported a diabetes diagnosed between 22 and 45 years of age, only 30% of which were taking insulin by the baseline examination. Without medical or medication histories, we were unable to validate if these were T2D, or Latent Autoimmune Diabetes in Adults cases who would be expected to be leaner on average [50]. Nonetheless we take confidence in the observation that the association of *TCF7L2* T2D-risk alleles and BMI was stronger among those without previous T2D diagnosis. In fact, individuals with impaired glucose tolerance and undiagnosed diabetes at examination had the greatest protective effect on BMI of T2D-risk alleles at *TCF7L2*. Forthcoming HCHS/SOL, or other prospective cohort follow up data will allow future investigators to explore the contemporaneous and interacting relationships between *TCF7L2,* BMI and T2D status across the adult life course.

Similarly, our structural equation modeling was notably limited by its reliance on self-reported weight histories, and height measured at the baseline examination to approximate the BMIs at 21, 45 and 65 years of age. Nonetheless this study cohort self-reported their current weight with good accuracy and reliability at baseline [34], and we robustly replicated our unstructured *TCF7L2* associations with BMI at examination (21–76 years) using both measured and self-reported current weights. Lastly, we cannot rule out the role of birth cohort or healthy immigrant effects in shaping the characteristics of our sample of predominantly foreign-born middle-aged adults, especially among the subset of older adults in HCHS/SOL (e.g. ≥65 years of age) who were healthy enough to be community-dwelling at the time of recruitment, and willing to participate in the extensive baseline examination. Our structured modeling sheds light on this survival bias, as T2D-risk alleles were non-significantly associated with an increased BMI at 65 years of age, independent of earlier BMIs and T2D statuses.

## Conclusions

Our significant population-based associations between T2D risk alleles at *TCF7L2* (rs7903146) and lower BMI do not support selection bias as the sole explanation of the *TCF7L2*-BMI association. This work contributes to a mounting body of literature reporting consistent protective effects of T2D risk alleles at *TCF7L2* and BMI, which points to a complex mechanistic structure underlying the functional consequences of *TCF7L2* on both T2D and BMI. Yet, future functional work is needed to describe the specific cell or tissue types that are most relevant to the observed *TCF7L2* action. Observational analyses may be particularly useful for estimating causal effects at this genetic locus and pinpointing windows of susceptibility for future public health interventions in populations, like US Hispanic/Latinos, which carry disproportionate burdens of both T2D and obesity.

### Additional file

Additional file 1: **Figure S1.** LDlink plot showing the regional r^2^ patterning between 1000 Genomes AMR and EUR reference populations at *TCF7L2* around rs7903146 (shown in blue) and rs12255372 (alternative marker of the rs7903146 signal), with the bubble size representing the frequency of each SNP and the support for each SNP’s regulatory potential shown numerically (strong to weak: 1–7 RegulomeDB Scores). **Table S1** Staged data cleaning and outlier identification on total sample of 40,525 self-reported weights from 16,355 adult participants (18–76 years) in the Hispanic Community Health Study/Study of Latinos (HCHS/SOL). **Figure S2.** Flow chart of staged quality control on 16,355 adult Hispanic/Latino participants (18–76 years) with at least one self-reported weight, as part of the anthropometric exam or weight history questionnaire, at the baseline examination (2008–2011) of the Hispanic Community Health Study/Study of Latinos (HCHS/SOL), resulting in 54 self-reported weights recoded due to unit confusion, 541 individuals excluded, and a final analytic sample of 16,322 participants. **Table S2.** Parameter Estimates of the Effect per Type 2 Diabetes Risk Allele (rs7903146-T) in a Multivariable Model Using Measured and Self-Reported Weight Measurements in the Analytic Sample Stratified by Diabetes Status at Baseline Examination and Medication. **Figure S3.** Illustration of all possible pathways in Structural Equation Model. **Table S3.** Parameter Estimates from Pathway Model Results. **Table S4.** Parameter Estimates for Select Indeirect Pathway Model Results. (DOCX 787 kb)

## Appendix

Between 2008 and 2011, up to 15 participants per site were invited to repeat the Hispanic Community Health Study/Study of Latinos (HCHS/SOL) examination, including the weight history questionnaire, one to three months after their initial baseline examination without additional reimbursement of their time (*N* = 56). A total of 52 participants reported body weights a median of 40 days after their initial baseline examination (range: 0 to 107 days). The mean differences of the 21 and 45-year old self-reported weight were − 0.9 kg (− 2.7, 1.0) and − 1.3 kg (− 3.8, 1.1) resulting in good reliabilities and low coefficients of variation (21-year recall: ICC = 0.89, CV = 7.7% *n* = 52; 45-year recall: 0.86, 6.7%, *n* = 29). Only two participants, ≥65 years of age, recalled their 65-year old weight twice, which precluded reliability calculations for 65-year old weights.

HCHS/SOL personnel (requiring a minimum of 5 practice subjects with 0.5 kg weight and 0.5 cm height agreement between a trainee and expert) measured height (cm) and weight (kg) during the anthropometric examination of the full HCHS/SOL cohort using a fixed (wall mounted) stadiometer (inspected daily) and a digital scale (scales zero balanced daily and calibrated weekly) on all participants that were able to stand on both feet while wearing a scrub suit or examination gown and no shoes [34]. Currently pregnant women were rescheduled for the HCHS/SOL baseline examination approximately 3 months after their delivery. The validity of self-reported weight, and inter-rater reliability of self-reported weight, measured weight and height have been reported to be good for the HCHS/SOL baseline examination [34].

HCHS/SOL study personnel were centrally trained and periodically observed. In addition we applied a data quality control protocol, as described previously for self-reported current weight collected as part of the anthropometric examination [34] to 1) minimize potential instances of unit confusion in the self-report (lb versus kg) or 2) exclude self-reported weights during pregnancy reported during the baseline examination, self-reported or measured weights made by individuals with past limb amputation or when scaled by measured baseline height that correspond to extreme underweight (< 16 kg/m^2^) or obesity (> 70 kg/m^2^). However, herein, we extended this protocol to also include recalled weights at 21, 45, or 65 years of age. We first flagged entire weight histories with either a ≥ 15 kg difference between current self-reported and measured weight, or at least two ≥15 kg pairwise fluctuations in weight (Additional file 1: Figure S1 and Table S1). Of the entire set of 16,355 individuals with at least one self-reported weight in adulthood (40,525 observations) and based on our staged quality control protocol, we recoded a total of 54 observations, and excluded an additional 541 observations (Additional file 1: Table S1). Only six self-reported weights from the anthropometric examination (age range 27–64 years) were recoded and retained in our final analytic sample.

## Abbreviations

BMI: Body Mass Index
GWAS: Genome-wide Association Study
HCHS/SOL: Hispanic Community Health Study/Study of Latinos
OGTT: Oral Glucose Tolerance Test
HbA1C: Glycated Hemoglobin
SNP: Single Nucleotide Polymorphism
T2D: Type 2 Diabetes
*TCF7L2*: Transcription Factor 7-like 2
US: United States
