# Supplementary material for: Complex patterns of direct and indirect association between the transcription Factor-7 like 2 gene, body mass index and type 2 diabetes diagnosis in adulthood in the Hispanic Community Health Study/Study of Latinos
Source: BMC Obes. 2018 Oct 2;5:26. doi: 10.1186/s40608-018-0200-x (PMC6167893; doi:10.1186/s40608-018-0200-x)
Supplement: Supplementary file 1 — Figure S1. LDlink plot showing the regional r2 patterning between 1000 Genomes AMR and EUR reference populations at TCF7L2 around rs7903146 (shown in blue) and rs12255372 (alternative marker of the rs7903146 signal), with the bubble size representing the frequency of each SNP and the support for each SNP’s regulatory potential shown numerically (strong to weak: 1–7 RegulomeDB Scores). Table S1 Staged data cleaning and outlier identification on total sample of 40,525 self-reported weights from 16,355 adult participants (18–76 years) in the Hispanic Community Health Study/Study of Latinos (HCHS/SOL). Figure S2. Flow chart of staged quality control on 16,355 adult Hispanic/Latino participants (18–76 years) with at least one self-reported weight, as part of the anthropometric exam or weight history questionnaire, at the baseline examination (2008–2011) of the Hispanic Community Health Study/Study of Latinos (HCHS/SOL), resulting in 54 self-reported weights recoded due to unit confusion, 541 individuals excluded, and a final analytic sample of 16,322 participants. Table S2. Parameter Estimates of the Effect per Type 2 Diabetes Risk Allele (rs7903146-T) in a Multivariable Model Using Measured and Self-Reported Weight Measurements in the Analytic Sample Stratified by Diabetes Status at Baseline Examination and Medication. Figure S3. Illustration of all possible pathways in Structural Equation Model. Table S3. Parameter Estimates from Pathway Model Results. Table S4. Parameter Estimates for Select Indeirect Pathway Model Results. (DOCX 787 kb) [file 40608_2018_200_MOESM1_ESM.docx]

**
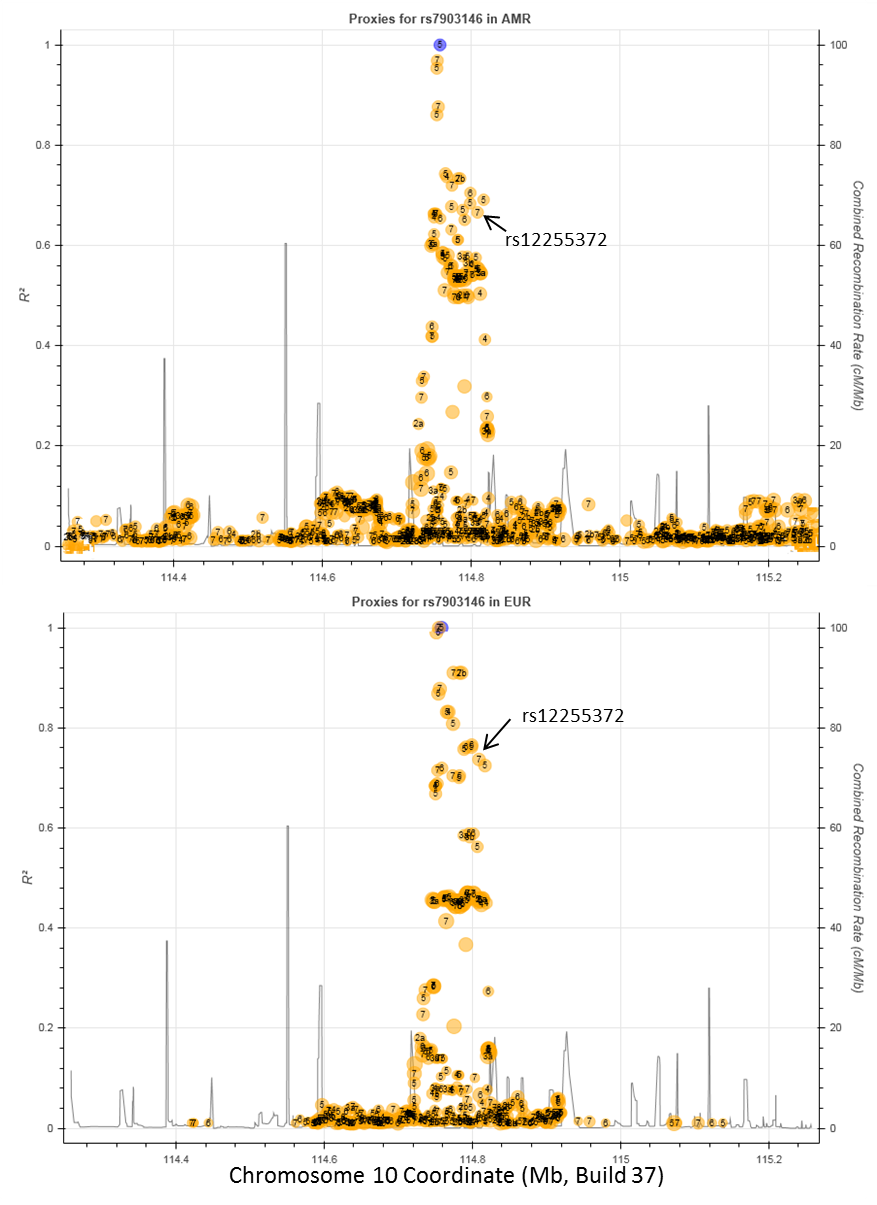
**

**Figure S1.** LDlink plot showing the regional r^2^ patterning between 1000 Genomes AMR and EUR reference populations at *TCF7L2* around rs7903146 (shown in blue) and rs12255372 (alternative marker of the rs7903146 signal), with the bubble size representing the frequency of each SNP and the support for each SNP’s regulatory potential shown numerically (strong to weak: 1-7 RegulomeDB Scores).

**Table S1.** Staged data cleaning and outlier identification on total sample of 40,525 self-reported weights from 16,355 adult participants (18-76 years) in the Hispanic Community Health Study/Study of Latinos (HCHS/SOL).

| **Action** | **Measure** | | | **Criteria** | **No. Observations Remaining** | **No. Individuals^a^**  **Remaining** |
| --- | --- | --- | --- | --- | --- | --- |
| **Stage 1- Iterative Data Cleaning of Flagged SR Weight Histories (≥15kg change)^b^** | | | | | | |
| Recoded^c^ | Current SR | ≥15kg decrease in change with current M weight | | | 40,525 (16 recoded^c^) | 16,355 |
| Excluded | Current SR | >2 SD^b^, ≥15kg change with current M weight | | | 40,484 | 16,351 |
| Recoded^c^ | 21, 45, 65 year | Plausible^d^, ≥15kg/decade decrease in fluctuation across ≥3 weights | | | 40,484 (38 recoded^c^) | 16,351 |
| Excluded | 21, 45, 65 year | >2 SD^b^, ≥15kg/decade change with current M weight | | | 40,209 | 16,351 |
| Excluded | 21, 45, 65 year, Current SR | <34.5kg | | | 40,190 | 16,349 |
|  |  | >200.8kg | | | 40,186 | 16,346 |
| **Stage 2- Outlier Identification of SR Weights** | | | | | | |
| Excluded | Current pregnancy^e^ | | Yes | | 40,172 | 16,345 |
|  | Previous limb amputation^e^ | | Yes | | 40,129 | 16,331 |
|  | BMI^f^ | | <16.0kg/m^2^ | | 39,984 | 16,322 |
|  |  | | >70.0kg/m^2^ | | - | - |
| **Final Analytic Sample of SR Weights** | | | | | **39,984** | **16,322** |

Abbreviations: BMI=Body mass index, No.=Number, SD=standard deviation, SR=self-reported, M=measured

^a^Because individuals can contribute multiple observations, the number of observations and individuals remaining after each step are shown separately.

^b^Specific questionable weights within flagged weight histories were those included in at least two ≥15kg changes, or those >2 standard deviations from the gender and age-specific mean (categories of age: 18-21, 22-29, 30-39, 40-49, 50-59, 60-69, 70-76 years). Across 6,990 flagged SR weight histories, 7,817 SR weights were identified as being questionable at the beginning of Stage 1. After completing Stage 1, 6,805 weight histories were flagged but represented the same or less questionable SR weights than prior to cleaning (7,460 questionable weights). No new flags were identified after the recoding of 54 observations in stage 1.

^c^The two possible scenarios of kg/lb SR were assessed (1- true SRs in kg were recorded as lb, 2- true SR in lb were recorded as kg) and the weight was recoded if one of the scenarios were favored according to the listed criteria.

^d^Recoded if the given scenario resulted in a weight within 2 standard deviations of the gender and age-specific mean (defined in footnote b) and was biologically plausible for height (18.5-50.0kg/m^2^, based on the 1^st^ to 99^th^ percentiles of M BMI at baseline for cohort, 18-76 years of age), provided that the participant’s BMI was in this range at baseline.

^e^Only current SR and M weights were excluded for women reporting to be currently pregnant in the medical history portion of the baseline examination, whereas all SR and M weights were excluded for individuals with a limb amputation at the baseline examination. M weights are not tallied in this table, but correspond to 14 excluded due to current pregnancy and 12 due to limb amputation.

^f^BMI was calculated for all SR weights using an individual’s measured adult height at the baseline examination, under the assumption that this would be static across adulthood. Although not included here, 10 M weights were identified as outliers using this exclusion.
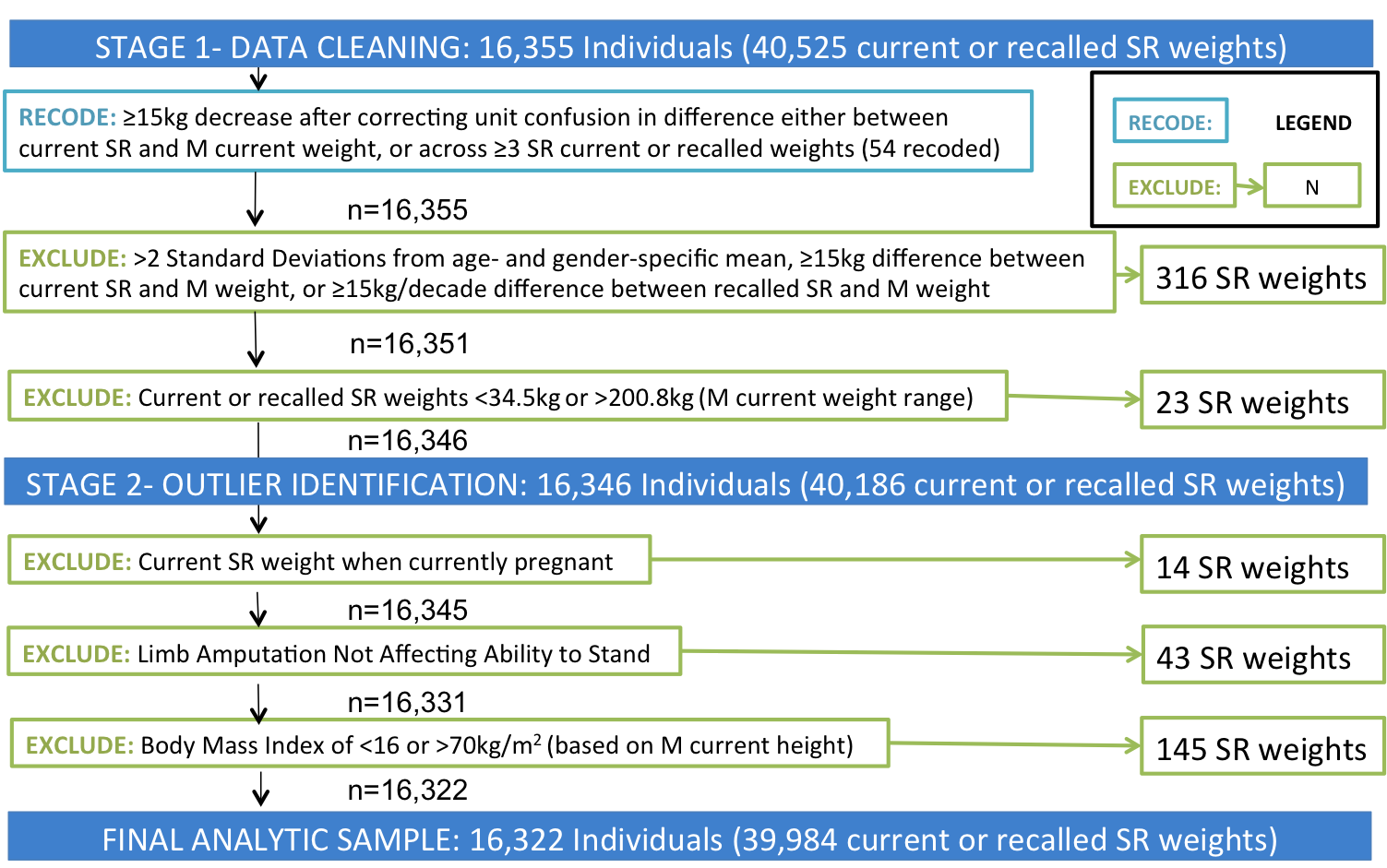


**Figure S2**. Flow chart of staged quality control on 16,355 adult Hispanic/Latino participants (18-76 years) with at least one self-reported weight, as part of the anthropometric exam or weight history questionnaire, at the baseline examination (2008-2011) of the Hispanic Community Health Study/Study of Latinos (HCHS/SOL), resulting in 54 self-reported weights recoded due to unit confusion, 541 individuals excluded, and a final analytic sample of 16,322 participants

|  |  | Outcome Measure | Measured BMI at Examination | Self-Reported BMI at Examination |
| --- | --- | --- | --- | --- |
| Individuals Without Diabetes Diagnosis Prior to Examination | Overall | Unweighted N* | N = 7,819 | N = 7,744 |
|  |  | Beta (95% CI) | -0.45  (-0.79, -0.10) | -0.48  (-0.82, -0.14) |
|  | Impaired Glucose Tolerance at Examination | Unweighted N* | N = 3,613 | N = 3,571 |
|  |  | Beta (95% CI) | -0.66  (-1.05, -0.26) | -0.70  (-1.08, -0.31) |
|  | Undiagnosed Diabetic at Examination** | Unweighted N* | N = 671 | N = 661 |
|  |  | Beta (95% CI) | -0.82  (-1.62, -0.02) | -0.82  (-1.56, -0.08) |
| Individuals With Diabetes Diagnosis Prior to Examination | Overall | Unweighted N* | N = 1,193 | N = 1,177 |
|  |  | Beta (95% CI) | -0.30  (-1.04, 0.43) | -0.17  (-0.93, 0.59) |
|  | Taking Medication(s) at Examination | Unweighted N* | N = 1,005 | N = 992 |
|  |  | Beta (95% CI) | -0.41  (-1.25, 0.44) | -0.23  (-1.09, 0.63) |

**Table S2. Parameter Estimates of the Effect per Type 2 Diabetes Risk Allele (rs7903146-T) in a Multivariable Model Using Measured and Self-Reported Weight Measurements in the Analytic Sample Stratified by Diabetes Status at Baseline Examination and Medication**

*Unweighted sample size differences were a result of individuals missing certain weight measurements including specifically for self-reported recall weights only individuals that were had reached those ages and had non-missing self-recall weights provided for that particular age

**Of the individuals who did not report a previous diabetes diagnosis and were classified as diabetic during the examination, only 5.2% of these participants (or 4.7% weighted for sampling design) were taking diabetes medications for high blood sugar or diabetes.


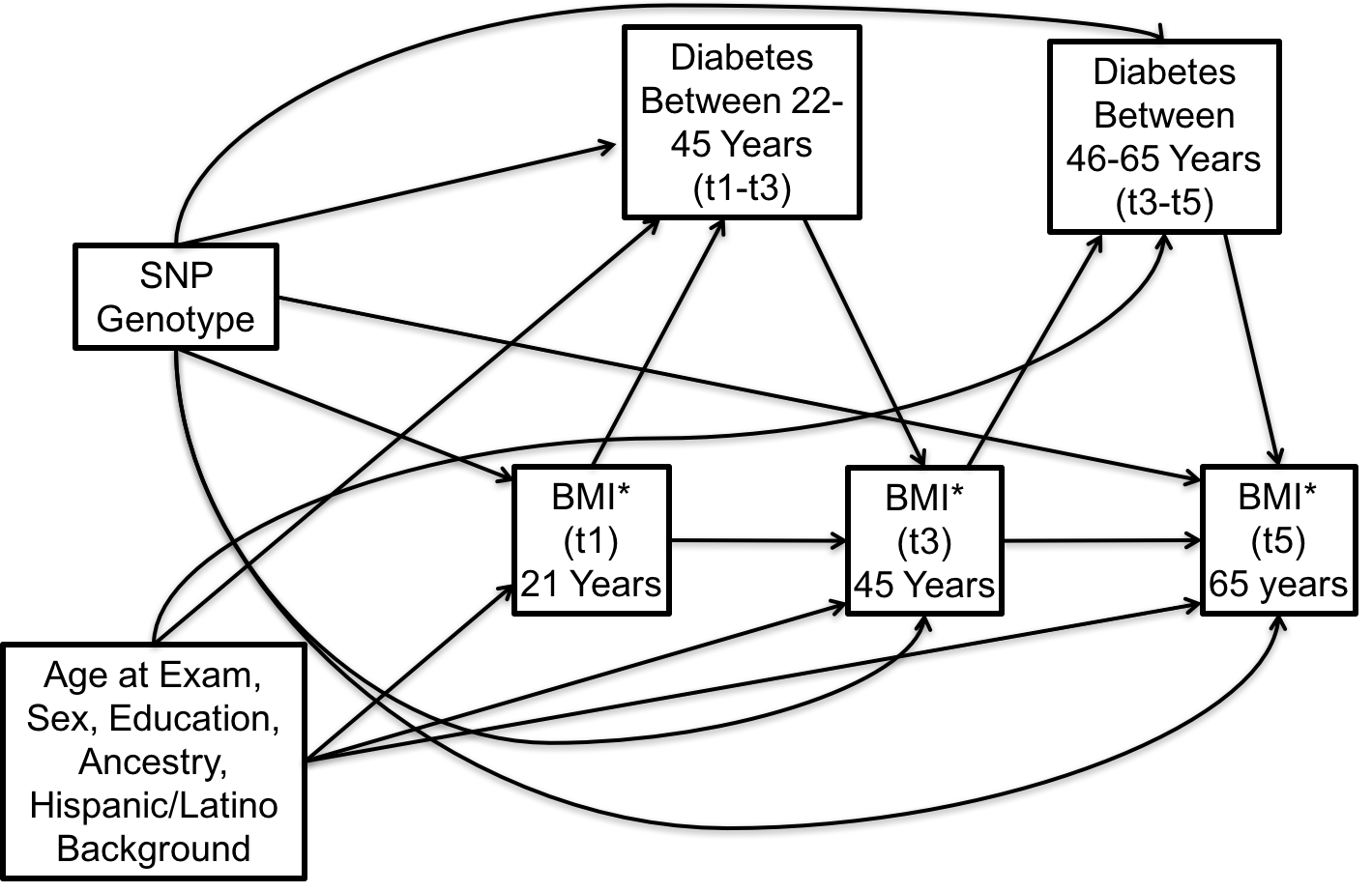


**Figure S3.** Illustration of all possible pathways in Structural Equation Model

| ***Table S3: Parameter Estimates from Pathway Model Results*** | | | | | |
| --- | --- | --- | --- | --- | --- |
| ***Outcome*** | ***Covariate*** | | | ***Parameter Estimates*** | ***95% CI*** |
| *BMI* | *At Age 21* | *rs7903146* |  | -0.21 | (-0.46, 0.04) |
|  |  | *Age at Baseline* |  | -0.07 | (-0.08, -0.05) |
|  |  | *Male* |  | 0.59 | (0.30, 0.87) |
|  |  | *Has High School diploma or GED* |  | -0.48 | (-0.81, -0.15) |
|  |  | *Ancestry* | *African* | 0.34 | (-0.71, 1.40) |
|  |  |  | *European* | Ref | Ref |
|  |  |  | *Southern Native American* | -1.86 | (-5.56, 1.83) |
|  |  |  | *Northern Native American* | 0.51 | (-1.05, 2.07) |
|  |  | *Hispanic Background* | *Central American* | -0.72 | (-1.69, 0.24) |
|  |  |  | *Cuban* | -0.97 | (-1.74, -0.20) |
|  |  |  | *Dominican* | -1.06 | (-2.13, 0.01) |
|  |  |  | *Mexican* | Ref | Ref |
|  |  |  | *Puerto Rican* | 0.42 | (-0.82, 1.66) |
|  |  |  | *South American* | -0.28 | (-1.50, 0.94) |
|  | *At Age 45* | *rs7903146* |  | -0.08 | (-0.29, 0.12) |
|  |  | *BMI at Age 21* |  | 0.52 | (0.46, 0.58) |
|  |  | *Diabetes Between Ages 22 and 45* |  | 0.57 | (-0.13, 1.27) |
|  |  | *Age at Baseline* |  | -0.11 | (-0.13, -0.08) |
|  |  | *Male* |  | -0.61 | (-0.90, -0.32) |
|  |  | *Has High School diploma or GED* |  | 0.05 | (-0.25, 0.35) |
|  |  | *Ancestry* | *African* | 0.17 | (-0.88, 1.21) |
|  |  |  | *European* | Ref | Ref |
|  |  |  | *Southern Native American* | -1.15 | (-4.20, 1.90) |
|  |  |  | *Northern Native American* | 1.22 | (-0.74, 3.17) |
|  |  | *Hispanic Background* | *Central American* | 0.48 | (-0.42, 1.38) |
|  |  |  | *Cuban* | 0.03 | (-0.75, 0.81) |
|  |  |  | *Dominican* | 0.37 | (-0.61, 1.35) |
|  |  |  | *Mexican* | Ref | Ref |
|  |  |  | *Puerto Rican* | 1.17 | (0.07, 2.27) |
|  |  |  | *South American* | -0.06 | (-1.20, 1.08) |
|  | *At Age 65* | *rs7903146* |  | 0.20 | (-0.41, 0.81) |
|  |  | *BMI at Age 45* |  | 0.79 | (0.64, 0.93) |
|  |  | *Diabetes Between Ages 46 and 65* |  | 0.46 | (-0.52, 1.44) |
|  |  | *Age at Baseline* |  | -0.13 | (-0.23, -0.03) |
|  |  | *Male* |  | -0.55 | (-1.26, 0.15) |
|  |  | *Has High School diploma or GED* |  | -0.26 | (-0.95, 0.44) |
|  |  | *Ancestry* | *African* | 0.32 | (-1.56, 2.20) |
|  |  |  | *European* | Ref | Ref |
|  |  |  | *Southern Native American* | -1.15 | (-7.68, 5.38) |
|  |  |  | *Northern Native American* | -4.97 | (-8.88, -1.06) |
|  |  | *Hispanic Background* | *Central American* | -0.10 | (-1.90, 1.69) |
|  |  |  | *Cuban* | -1.15 | (-2.72, 0.43) |
|  |  |  | *Dominican* | -1.96 | (-3.98, 0.06) |
|  |  |  | *Mexican* | Ref | Ref |
|  |  |  | *Puerto Rican* | -1.00 | (-3.03, 1.04) |
|  |  |  | *South American* | -1.09 | (-3.38, 1.19) |
| *T2D* | *Between Ages 22 and 45* | *rs7903146* |  | 0.28 | (0.04, 0.51) |
|  |  | *BMI at Age 21* |  | 0.11 | (0.08, 0.14) |
|  |  | *Age at Baseline* |  | -0.04 | (-0.06, -0.01) |
|  |  | *Male* |  | -0.05 | (-0.35, 0.26) |
|  |  | *Has High School diploma or GED* |  | -0.38 | (-0.71, -0.04) |
|  |  | *Ancestry* | *African* | 0.08 | (-1.07, 1.24) |
|  |  |  | *European* | Ref | Ref |
|  |  |  |  |  |  |
|  |  |  | *Southern Native American* | 0.87 | (-2.00, 3.74) |
|  |  |  | *Northern Native American* | 0.73 | (-1.16, 2.62) |
|  |  | *Hispanic Background* | *Central American* | -0.34 | (-1.24, 0.56) |
|  |  |  | *Cuban* | -0.4 | (-1.35, 0.56) |
|  |  |  | *Dominican* | 0.43 | (-0.61, 1.47) |
|  |  |  | *Mexican* | Ref | Ref |
|  |  |  | *Puerto Rican* | -0.02 | (-1.17, 1.14) |
|  |  |  | *South American* | -1.05 | (-2.17, 0.07) |
|  | *Between Ages 46 and 65* | *rs7903146* |  | 0.51 | (0.13, 0.89) |
|  |  | *BMI at Age 45* |  | 0.17 | (0.09, 0.25) |
|  |  |  |  |  |  |
|  |  | *Age at Baseline* |  | -0.11 | (-0.21, -0.02) |
|  |  | *Male* |  | 0.33 | (-0.10, 0.77) |
|  |  | *Has High School diploma or GED* |  | -0.05 | (-0.54, 0.45) |
|  |  | *Ancestry* | *African* | 0.45 | (-0.98, 1.88) |
|  |  |  | *European* | Ref | Ref |
|  |  |  | *Southern Native American* | -0.93 | (-5.60, 3.74) |
|  |  |  | *Northern Native American* | 4.7 | (1.62, 7.77) |
|  |  | *Hispanic Background* | *Central American* | -0.15 | (-1.70, 1.40) |
|  |  |  | *Cuban* | 1.67 | (0.31, 3.02) |
|  |  |  | *Dominican* | 2.26 | (0.68, 3.83) |
|  |  |  | *Mexican* | Ref | Ref |
|  |  |  | *Puerto Rican* | 1.71 | (0.06, 3.35) |
|  |  |  | *South American* | 0.84 | (-1.06, 2.73) |

| ***Table S4: Parameter Estimates for Select Indeirect Pathway Model Results*** | | | |
| --- | --- | --- | --- |
| ***Exposures to*** ***Intermediates*** | ***Outcome*** | ***Parameter Estimates*** | ***95% CI*** |
| *SNP to BMI at 21 to Diabetes from 21-44* | *BMI at 45 years* | -0.013 | (-0.033, 0.007) |
| *SNP to BMI at 21 to BMI at 45 to Diabetes from 45-64* | *BMI at 65 years* | -0.009 | (-0.031, 0.013) |
| *SNP to BMI at 21 to Diabetes from 21-44 to BMI at 45 to Diabetes between 45-64* | *BMI at 65 years* | -0.001 | (-0.003, 0.001) |
